# Adjusted D-dimer cutoff levels to rule out pulmonary embolism in patients hospitalized for COPD exacerbation: results from the SLICE trial

**DOI:** 10.1186/s12959-022-00368-0

**Published:** 2022-03-03

**Authors:** Carmen Rodríguez, Luis Jara-Palomares, Eva Tabernero, Andrés Tenes, Sara González, Winnifer Briceño, José Luis Lobo, Raquel Morillo, Behnood Bikdeli, David Jiménez

**Affiliations:** 1 Respiratory Department, Hospital Ramón y Cajal and Instituto Ramón y Cajal de Investigación Sanitaria IRYCIS, Colmenar Road, Km. 9,100, 28034 Madrid, Spain; 2 Respiratory Department, Virgen del Rocío Hospital and Instituto de Biomedicina, Sevilla, Spain; 3 CIBER Enfermedades Respiratorias (CIBERES), Madrid, Spain; 4 Respiratory Department, Hospital Universitario Cruces, Biocruces-Bizkaia, Barakaldo, Spain; 5 Respiratory Department, Hospital Araba, Vitoria, Spain; 6 Cardiovascular Medicine Division, Brigham and Women’s Hospital, Harvard Medical School, Boston, MA USA; 7 Center for Outcomes Research and Evaluation (CORE), Yale University School of Medicine, New Haven, CT USA; 8 Cardiovascular Research Foundation, New York, USA; 9 Medicine Department, Universidad de Alcalá, Madrid, Spain

**Keywords:** Pulmonary embolism, COPD, Clinical probability, Diagnosis, D-dimer

## Abstract

**Background:**

For patients with suspected pulmonary embolism (PE), age- or clinically-adjusted D-dimer threshold level can be used to define a negative test that safely excludes PE and reduces the use of imaging. However, the utility of this approach in patients hospitalized for chronic obstructive pulmonary disease (COPD) exacerbation is undefined.

**Methods:**

We ran an analysis of the patients hospitalized for COPD exacerbation and randomized to the intervention in the SLICE trial. Using the conventional strategy as the reference, *we* compared the proportion of patients with a negative D-dimer result, and the negative predictive value and sensitivity of three D-dimer threshold strategies for initial PE or subsequent diagnosis of venous thromboembolism (VTE): the age-adjusted strategy, the Wells-adjusted strategy, and the YEARS-adjusted strategy.

**Results:**

We included 368 patients. Using a conventional threshold, 182 (49.5%) patients had negative D-dimer values, of whom 1 (0.6%) had PE (sensitivity, 94.1%). The use of an age-adjusted threshold increased the number of patients in whom PE could be excluded from 182 to 233 patients (63.3%), and the proportion of false-negative findings increased from 0.5% to 1.7% (sensitivity, 76.5%). With the use of the Wells or YEARS strategies, 64.4% and 71.5% had negative values, and the proportion of false-negative findings was 2.5% (sensitivity, 64.7%) and 2.7% (sensitivity, 58.8%), respectively.

**Conclusions:**

In patients hospitalized for COPD exacerbation, compared with the conventional strategy, age- or clinically-adjusted strategies of D-dimer interpretation were associated with a larger proportion of patients in whom PE was ruled out with a higher failure rate.

**Trial Registration:**

ClinicalTrials.gov number: NCT02238639.

## Introduction

D-dimer is the initial diagnostic test for patients with suspected acute symptomatic pulmonary embolism (**PE**) [1, 2]. In patients with low or intermediate clinical pretest probability, a normal D-dimer test result safely excludes acute PE without the need for imaging testing [3]. In order to increase the clinical usefulness of D-dimer testing (i.e., to reduce the need for computed tomography pulmonary angiography [**CTPA**]) without missing relevant PE, several strategies for revising the conventional fixed D-dimer threshold have been proposed. These strategies include age-adjusted D-dimer interpretation and clinical probability-adjusted interpretation of D-dimer.

Studies have shown that these strategies can be effective and safe in the exclusion of PE and in the reduction of the risk of false positives when compared to the conventional D-Dimer cutoff strategy. The age-adjusted strategy for D-dimer interpretation uses a progressively higher D-dimer threshold to categorize results as positive in patients over 50 years of age (i.e., use of age multiplied by 10) [4]. A second strategy consists of ruling out PE in patients with a low clinical pretest probability (according to the Wells score) and a D-dimer level of less than 1000 ng/mL, and in those with a moderate clinical pretest probability and a D-dimer level of less than 500 ng/mL [5]. Finally, the YEARS diagnostic algorithm uses twice the D-dimer threshold (i.e., 1000 ng/mL) to exclude PE in patients with no YEARS criteria (clinical signs of deep vein thrombosis [**DVT**], hemoptysis, and PE as the most likely diagnosis), and a threshold of 500 ng/mL in patients with one or more criteria [6].

Though PE has been reported to be prevalent in patients with exacerbation of chronic obstructive pulmonary disease (**COPD**) [7–10], the Significance of Pulmonary Embolism in COPD Exacerbations (**SLICE**) showed that an active strategy for the diagnosis of PE (D-dimer testing and, if positive, CTPA) did not result in a lower percentage of patients experiencing the composite outcome of venous thromboembolism (**VTE**), readmission for COPD, or death within 90 days after randomization [11]. In daily practice, however, D-dimer testing is frequently ordered for the initial assessment of the patients presenting with chest symptoms (including those with COPD exacerbations). Such testing is, in many cases, unnecessary.

The objective of this study was to assess the clinical usefulness and diagnostic accuracy of the age-adjusted and clinical probability-adjusted strategies of D-dimer interpretation, compared with the conventional fixed threshold, in a large group of patients hospitalized for exacerbations of COPD.

## Methods

We performed post hoc analyses of the recently completed SLICE trial. SLICE was a multicenter, open-label, randomized, clinical trial aimed at evaluating whether an active search for PE might improve clinical outcomes in patients with exacerbations of COPD who required hospital admission. The rationale, design and main results of the SLICE study were described previously [11, 12]. The trial was conducted in 18 academic hospitals across Spain. The institutional review board at each of the participating sites approved the protocol, and each patient provided written informed consent.

Briefly, consecutive patients with exacerbations of COPD who required hospital admission and had no initial clinical suspicion of PE were randomized to either the usual care plus an active search for PE (intervention group) or usual care alone (control group). Patients in the intervention group were investigated with a sequential diagnostic strategy based on D-dimer testing and CTPA. We used the Fibrinogen Equivalent Unit (**FEU**) to report D-dimer levels based on the molecular weight of fibrinogen. For patients with a negative conventional D-dimer (defined by the department of clinical chemistry at each participating site [Table 4 in the Appendix]), a diagnosis of PE was deemed as ruled out by the treating clinicians. For patients with a positive D-dimer according to the conventional criteria, a CTPA was performed. CTPA results were categorized as positive for PE if an intraluminal filling defect was seen in subsegmental or more proximal branches, and were considered negative if no filling defect was observed. For the present analysis, only patients who were randomized to the intervention were included.

### D-dimer interpretation strategy definitions

#### Age-adjusted strategy

D-dimer results were categorized as negative if the D-dimer level was less than the conventional threshold used at each participating site in patients 50 years or younger or, in patients older than 50 years, if it was less than the patient’s age multiplied by 10 (e.g., less than 650 ng/mL if 65 years; less than 800 ng/mL if 80 years) [4].

#### Clinical probability-adjusted strategy

According to the seven-item Wells clinical prediction rule [5], D-dimer results were categorized as negative if the D-dimer level was less than 1000 ng/mL in patients with low clinical pretest probability and less than 500 ng/mL for patients with moderate clinical pretest probability.

The YEARS diagnostic algorithm consists of clinical signs of DVT, hemoptysis, and PE as the most likely diagnosis [6]. D-dimer results were categorized as negative if the D-dimer level was less than 1000 ng/mL in patients without any YEARS item and less than 500 ng/mL for patients with one or more of the YEARS items.

### Outcome measures

The primary outcome for this analysis was the rate of adjudicated PE events at initial testing, or VTE events within a 3-month follow-up period in patients who did not receive anticoagulant therapy on the basis of negative results on the initial work-up.

A central independent adjudication committee whose members were unaware of management allocation adjudicated all suspected events during the study period.

### Statistical analysis

A 2 x 2 table was constructed for each strategy according to whether the D-dimer result in individual patients was categorized as positive or negative, and whether the patient was categorized as VTE-positive or VTE-negative. Estimates of the proportion of all patients who had a negative D-dimer test, sensitivity, specificity, and negative predictive value (**NPV**) were calculated for all strategies, along with 95% confidence intervals (**CI**) using the Wilson score method. Using the conventional strategy as the reference, pairwise comparisons of the proportion of all patients with negative D-dimer results, sensitivity, and specificity were performed using exact binomial testing, and 95% CIs for the absolute differences were calculated using the Agresti and Min approach [13]. For the NPV, the method for paired data proposed by Leisenring, Alonzo and Pepe was used (generalized score statistic) [14]. Comparisons were considered significant if the two-sided P-values were less than 0.05. All statistical analyses were performed with the use of the SPSS/PC software package (version 26, SPSS) and the DTComPair package in R version 3.2.3.

## Results

After excluding 1 patient who did not undergo D-dimer testing within 12 hours after randomization and 1 patient who had a high clinical pretest probability, a total of 368 patients were eligible for this analysis. The mean (SD) age was 70.2 (9.8) years, and 23.4% of the patients were women. A total of 44.3% of the patients had a low clinical pretest probability, and 55.7% had a moderate clinical pretest probability (Table 1). There were 2 PE-positive patients (1.2%) among those with low clinical pretest probability and 15 PE-positive patients (7.3%) among those with moderate clinical pretest probability.

**Table 1:** Baseline characteristics of the patients

Parameter	No. (%) of patients		
	Low clinical probability^a^ (*N*=163)	Intermediate clinical probability^b^ (*N*=205)	Total (*N*=368)
Age ≥75 years	63 (38.7)	69 (33.7)	132 (35.9)
Sex			
Male	124 (76.1)	158 (77.1)	282 (76.6)
Female	39 (23.9)	47 (22.9)	86 (23.4)
Current smoker	46 (28.2)	72 (35.1)	118 (32.1)
Pack-years of smoking, mean (SD), no.	58.2 (29.2)	59.5 (25.8)	59.0 (27.1)
COPD exacerbations in the past 12 months, mean (SD), no.	1.1 (1.6)	1.5 (1.9)	1.3 (1.8)
Very severe COPD: <30% of the predicted normal FEV_1_	18 (11.0)	37 (18.0)	55 (14.9)
Severe COPD: 30 to <50% of the predicted normal FEV_1_	70 (42.9)	99 (48.3)	169 (45.9)
Moderate COPD: 50 to <80% of the predicted normal FEV_1_	56 (34.4)	57 (27.8)	113 (30.7)
Mild COPD: ≥80% of the predicted normal FEV_1_	19 (11.7)	12 (5.9)	31 (8.4)
Risk factors for VTE			
Immobilization^c^	1 (0.6)	65 (31.7)	66 (17.9)
Cancer^d^	6 (3.7)	6 (2.9)	12 (3.3)
History of VTE	0 (0)	9 (4.4)	9 (1.3)
Surgery^e^	0 (0)	1 (0.5)	1 (0.3)
Obstructive sleep apnea	36 (22.1)	20 (9.8)	56 (15.2)
Congestive heart failure	25 (15.3)	23 (11.2)	48 (13.0)
Hormone therapy	3 (1.8)	2 (1.0)	5 (1.4)
Clinical symptoms and signs at presentation			
Dyspnea	163 (100)	203 (99.0)	366 (99.5)
Heart rate >100/min	0 (0)	127 (62.0)	127 (34.5)
Systolic blood pressure <100 mm Hg	3 (1.8)	8 (3.9)	11 (16.2)
Spo_2_ <90% (*N*=365)	62 (38.3)	83 (40.9)	145 (39.7)
Temperature ≥38°C	0 (0)	1 (0.5)	1 (0.3)
Increased sputum volume	59 (36.2)	69 (33.7)	128 (34.8)
Purulent sputum	11 (6.7)	13 (6.3)	24 (6.5)
Hemoptysis	2 (1.2)	1 (0.5)	3 (0.8)
Signs of DVT	0 (0)	0 (0)	0 (0)
Admission blood tests			
Creatinine >1.5 mg/dL	4 (2.5)	2 (1.0)	6 (1.6)
Hemoglobin, mean (SD), g/dL	14.4 (1.9)	14.1 (2.2)	14.3 (2.1)
Treatment for exacerbation			
Short-acting inhaled beta2-agonists	159 (97.5)	203 (99.0)	362 (98.4)
Short-acting inhaled anticholinergics	163 (100)	205 (100)	368 (100)
Systemic corticosteroids	135 (82.8)	165 (80.5)	300 (81.5)
Antibiotics	113 (69.3)	134 (65.4)	247 (67.1)
Pharmacological thromboprophylaxis (LMWH)	163 (100)	205 (100)	368 (100)

*Abbreviations: COPD* Chronic obstructive pulmonary disease, *SD* Standard deviation, *FEV*_*1*_ Forced expiratory volume in the first second, *VTE* Venous thromboembolism, *DVT* Deep vein thrombosis

^a^Wells score for PE less than 2 points

^**b**^Wells score for PE 2 to 6 points

^c^Immobilized patients defined as non-surgical patients who had been immobilized (i.e., total bed rest with bathroom privileges) for ≥4 days in the month prior to exacerbation of COPD

^d^Active or under treatment in the last year

^e^In the previous month

### Comparison of the strategies

The proportion of patients with a negative result was 49.5% with the conventional threshold strategy, 63.3% with the age-adjusted strategy (difference 13.8%; 95% CI, 6.5% to 21.0%; *P* <0.001), 64.4% with the Wells strategy (difference 14.9%; 95% CI, 7.6% to 22.1%; *P* <0.001), and 71.5% with the YEARS strategy (difference 22.0%; 95% CI, 14.8% to 28.9%; *P* <0.001).

Sensitivity of the conventional strategy was 94.1%, while it was 76.5% with the age-adjusted strategy (difference 17.6%; 95% CI, -11.8% to 44.9%; *P* =0.02), 64.7% with the Wells strategy (difference 29.4%; 95% CI, -2.5% to 56.1%; *P* <0.001), and 58.8% with the YEARS strategy (difference 35.3%; 95% CI, 2.3% to 61.3%; *P* <0.001). NPVs and negative likelihood ratios of the different strategies are shown in Table 2.

**Table 2:** Accuracy of D-dimer interpretation strategies for VTE

Parameter	Conventional strategy	Age-adjusted strategy	Wells-adjusted strategy	YEARS-adjusted strategy
Negative results				
n/N	182/368	233/368	237/368	368
% (95% CI)	49.5 (44.2-54.7)	63.3 (58.2-68.3)	64.4 (59.3-69.3)	71.5 (66.6-76.0)
Sensitivity				
n/N	16/17	13/17	11/17	10/17
% (95% CI)	94.1 (71.3-99.9)	76.5 (50.1-93.2)	64.7 (38.3-85.8)	58.8 (32.9-81.6)
Specificity				
n/N	181/351	229/351	231/351	256/351
% (95% CI)	51.6 (46.2-56.9)	65.2 (60.0-70.2)	65.8 (60.6-70.8)	72.9 (68.0-77.5)
Negative predictive value				
n/N	181/182	229/233	231/237	256/263
% (95% CI)	99.5 (97.0-100)	98.3 (95.7-99.5)	97.5 (94.6-99.1)	97.3 (94.6-98.9)
Negative likelihood ratio	0.11	0.36	0.54	0.56
% (95% CI)	1.66-2.29	0.15-0.85	0.28-1.03	0.32-1.00
Positive likelihood ratio	1.95	2.20	1.89	2.17
% (95% CI)	0.02-0.76	1.63-2.97	1.29-2.77	1.41-3.35

*Abbreviations: CI* Confidence interval

Categorization of D-dimer results as positive or negative by the conventional and the age-adjusted strategies was in agreement for 317 (86%) patients and in disagreement for 51 (14%) patients (Table 3). Of the disagreements, 51 patients were categorized as positive by the conventional strategy and negative by the age-adjusted strategy. There were 3 PE-positive patients among those who were D-dimer positive by one strategy and negative by the other. Agreements between the conventional strategy and the Wells and YEARS strategies are shown in Table 3.

**Table 3:** Comparison of the conventional strategy and the age-adjusted and clinical probability-adjusted strategies with prevalence of VTE according to agreement

		**Age-adjusted strategy**		
		**D-dimer negative**	**D-dimer positive**	**Total**
**Conventional strategy**	D-dimer negative	182 1 PE (0.5%)	0	182
	D-dimer positive	51 3 PE (5.9%)	135 13 PE (9.6%)	186
	Total	233	135	368
		**Wells-adjusted strategy**		
		**D-dimer negative**	**D-dimer positive**	**Total**
**Conventional strategy**	D-dimer negative	182 1 PE (0.5%)	0	182
	D-dimer positive	55 5 PE (9.1%)	131 11 PE (8.4%)	186
	Total	237	131	368
		**YEARS-adjusted strategy**		
		**D-dimer negative**	**D-dimer positive**	**Total**
**Conventional strategy**	D-dimer negative	182 1 PE (0.5%)	0	182
	D-dimer positive	81 6 PE (7.4%)	105 10 PE (9.5%)	186
	Total	263	105	368

### Findings according to clinical probability

**Fig. 1:**
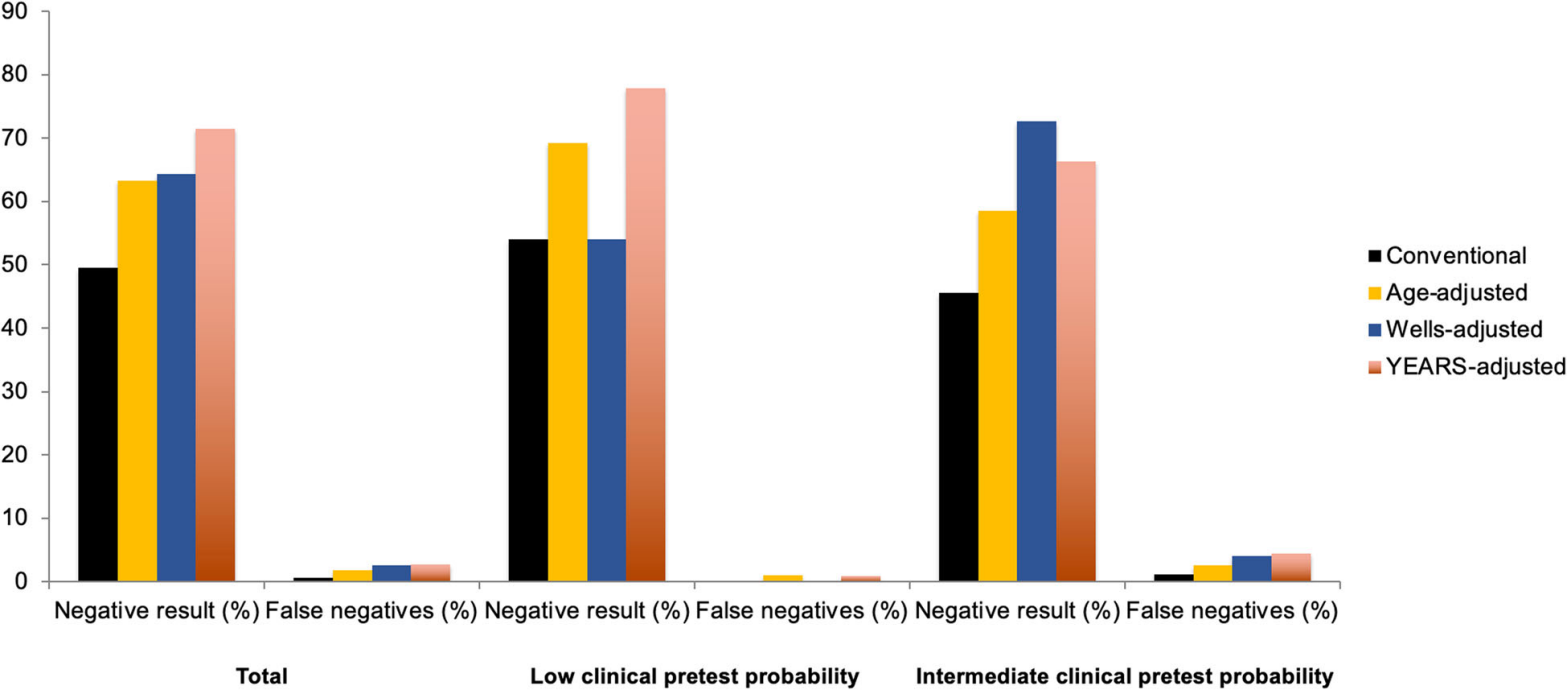
Proportion of patients with negative D-dimer results and with false negative results according to different D-dimer strategies. Clinical pretest probability according to the Wells score

According to the Wells score, 163 (44.3%) patients had low clinical pretest probability. The conventional D-dimer strategy categorized 88 (54.0%) patients with low clinical pretest probability as negative, and had a NPV of 100% (95% CI, 95.9% to 100%). The age-adjusted D-dimer strategy categorized 113 (69.3%) patients with low clinical pretest probability as negative, and had a NPV of 99.1% (95% CI, 95.2% to 100%). The Wells-adjusted D-dimer strategy categorized 88 (54.0%) patients with low clinical pretest probability as negative, and had a NPV of 100% (95% CI, 95.9% to 100%). The YEARS-adjusted D-dimer strategy categorized 127 (77.9%) patients with low clinical pretest probability as negative, and had a NPV of 99.2% (95% CI, 95.7% to 100%) (Fig. 1).

Of the total, 205 (55.7%) patients had moderate clinical probability. The conventional D-dimer strategy categorized 94 (45.6%) patients with moderate clinical pretest probability as negative, and had a NPV of 98.9% (95% CI, 94.2% to 100%). The age-adjusted D-dimer strategy categorized 120 (58.5%) patients with moderate clinical pretest probability as negative, and had a NPV of 97.5% (95% CI, 92.9% to 99.5%). The Wells-adjusted D-dimer strategy categorized 149 (72.7%) patients with moderate clinical pretest probability as negative, and had a NPV of 96.0% (95% CI, 91.4% to 98.5%). The YEARS-adjusted D-dimer strategy categorized 136 (66.3%) patients with moderate clinical pretest probability as negative, and had a NPV of 95.6% (95% CI, 90.6% to 98.4%) (Fig. 1)*.*

Overall, 311 (84.5%) patients had no YEARS items. The conventional D-dimer strategy categorized 164 (52.7%) patients with no YEARS items as negative, and had a NPV of 99.4% (95% CI, 96.7% to 100%). The age-adjusted D-dimer strategy categorized 212 (68.2%) patients with no YEARS items as negative, and had a NPV of 98.1% (95% CI, 95.2% to 99.5%). The Wells-adjusted D-dimer strategy categorized 206 (66.2%) patients with no YEARS items as negative, and had a NPV of 97.1% (95% CI, 93.8% to 98.9%). The YEARS-adjusted D-dimer strategy categorized 245 (78.8%) patients with no YEARS items as negative, and had a NPV of 97.1% (95% CI, 94.2% to 98.8%).

Among the 368 patients, 57 (15.5%) patients had one or more YEARS items. The conventional D-dimer strategy categorized 18 (31.6%) patients with one or more YEARS items as negative, and had a NPV of 100% (95% CI, 81.5% to 100%). The age-adjusted D-dimer strategy categorized 21 (36.8%) patients with one or more YEARS items as negative, and had a NPV of 100% (95% CI, 83.9% to 100%). The Wells-adjusted D-dimer strategy categorized 31 (54.4%) patients with one or more YEARS items as negative, and had a NPV of 100% (95% CI, 88.8% to 100%). The YEARS-adjusted D-dimer strategy categorized 18 (31.6%) patients with one or more YEARS items as negative, and had a NPV of 100% (95% CI, 81.5% to 100%).

## Discussion

Our study provides external validation of different strategies of D-dimer interpretation in patients hospitalized for COPD exacerbation and no initial clinical suspicion of PE. While the conventional strategy was able to exclude PE in one half of the patients, the use of age- or clinically-adjusted D-dimer threshold strategies resulted in an even higher percentage of patients in whom PE could be considered ruled out without the need for imaging. Both the conventional and age-adjusted strategies were associated with a low risk of PE diagnosed at initial testing or VTE within a 3-month follow-up period [15], but only the conventional fixed D-dimer threshold strategy had a sensitivity high enough to rule out PE.

Our results are in line with those observed in studies using an age-adjusted D-dimer threshold [4, 16, 17]. Particularly, for patients with COPD and a PE-unlikely Wells score, a systematic review and individual-patient data meta-analysis showed that age-adjusted D-dimer testing was effective (i.e., increased the proportion of COPD patients managed without imaging from 21% to 32%) and safe (i.e., the false negative rate increased from 0.7% to 1.2%) [18]. SLICE showed that the application of the age adjusted D-dimer cut-off value would result in the exclusion of VTE in almost 2 out of 3 (63.3%) of the patients hospitalized with COPD exacerbation, while the negative predictive value stayed above 98%. Although the magnitude of false negative findings seemed small, some caution may be warranted in using the age-adjusted strategy in settings with a higher PE prevalence, since its sensitivity was low.

Previous studies have shown that the clinical-adjusted strategies (i.e., the Wells score and the YEARS algorithm) have the potential to safely reduce CTPA use [5, 6]. Our analysis suggests that the conventional strategy is a better way of interpreting D-dimer results than the clinical probability-adjusted strategy among patients hospitalized with COPD exacerbation and without initial clinical suspicion of PE. Study design, setting and patient selection might account for the difference between previous studies and this analysis. Moreover, some data suggest that the accuracy of clinical prediction rules might be compromised in patients with underlying cardiopulmonary disease [19].

This study might have practical implications. The SLICE trial showed that an active diagnostic strategy for PE is not beneficial among patients hospitalized for an exacerbation of COPD [11]. However, D-dimer is often used in daily practice as a screening test in patients admitted to the Emergency Departments with chest symptoms [20]. On the basis of this analysis, clinicians might consider the use of a conventional fixed threshold interpretation for patients with COPD exacerbations who have a D-dimer test result because it avoids imaging in half of the patients with great safety. The efficiency is most pronounced in patients with low clinical pretest probability. Since patients with COPD exacerbations tend to be older, the age-adjusted strategy might be employed to reduce unnecessary exposure to radiation and potentially harmful contrast medium, unacceptable diagnosis-related and treatment-related costs, and serious or life-threatening bleeding complications of unjustified anticoagulation therapy.

### Limitations

This study has several limitations. First, this was a *post hoc* analysis of the intervention arm in a large randomized controlled trial with adjudicated outcomes. However, all the variables required to apply the Wells score and the YEARS algorithm were prospectively collected. Second, there was no a priori sample size calculation, and the modest number of patients in the analysis might not have provided estimates with reasonable precision. Third, the prevalence of PE was low. Therefore, the safety of the strategies might be lower in settings with a higher PE prevalence. Fourth, different D-dimer assays were used in the trial. However, we didn´t find any interaction between the D-dimer type or cutoff and the study results. Fifth, the findings in the study population might not apply to other patients with COPD. However, the inclusion and exclusion criteria were intended to be consistent with the pattern of patients admitted to the hospital with exacerbations of COPD. Finally, additional details about the body mass index, and baseline use of medications such as aspirin or statins would have been interesting. However, such data elements were not collected systematically in SLICE. This limitation is unlikely to undermine the findings of the current investigation.

## Conclusions

Among patients hospitalized for an exacerbation of COPD, our study suggests that the age- and clinically-adjusted strategies of D-dimer interpretation were associated with a larger number of patients in whom PE could be considered ruled out with a higher likelihood of subsequent PE than the conventional strategy. Although the magnitude of false negative findings with the age-adjusted strategy was small, additional studies are warranted to ascertain the safety of this approach.

## Data Availability

Data will be available upon reasonable request by Dr Jiménez, djimenez.hrc@gmail.com

## Appendix 1

**Investigators:** Barcelona (20 patients) – S. Jiménez (16 patients), A. Vilas (3 patients), D. Aisa (1 patient); Bilbao (113 patients) – E. Tabernero/B. González-Quero (113 patients); Galdakao (21 patients) – A. Ballaz/L. Chasco (21 patients); Gran Canaria (35 patients) – G. Pérez-Peñate/F. León-Marrero (35 patients); La Coruña (25 patients) – P. Marcos-Rodríguez/S.J. Domínguez-Pazos (25 patients); Madrid (364 patients) – D. Jiménez/A. Quezada (171 patients), A. Hernando/J.I. de Granda-Orive (91 patients), P. Ruiz-Artacho/F. Beddar-Chaib (56 patients), M.J. Rodríguez-Nieto/Itziar Fernández-Ormaechea (28 patients), M. Calle/J.L. Rodríguez-Hermosa/J. Carriel (12 patients), A. Martínez-Verdasco (2 patients), J. de Miguel-Díez (2 patients), M.A. Quesada (2 patients); Santander (17 patients) – R. Agüero (17 patients); Sevilla (112 patients) – L. Jara-Palomares/R. Otero/E. Márquez-Martín (112 patients); Valencia (8 patients) – R. López-Reyes (8 patients); Vitoria (31 patients) – J.L. Lobo/A. Rivas-Guerrero (31 patients).

## Appendix 2

Table 4 Methods for D-dimer analysis and cut-offs

**Table:**

Test	Criteria for positivity	Unit type
D-dimer ELISA KIT	>500 ng/mL	FEU
VIDAS D-dimer	>500 ng/mL	FEU
IL Test D-dimer	>500 ng/mL	FEU
HemosIL D-Dimer	>250 ng/mL	DDU
HemosIL AcuStar	>491 ng/mL	FEU
HemosIL D-dimer HS 500	>500 ng/mL	FEU
INNOVANCE D-dimer Assay	>500 ng/mL	FEU
STA-Liatest D-Di	>500 ng/mL	FEU

*Abbreviations: FEU* Fibrinogen Equivalent Unit, *DDU* D-Dimer Unit

## Abbreviations

CI: confidence interval
COPD: Chronic obstructive pulmonary disease
CTPA: Computed tomography pulmonary angiography
DDU: D-dimer Unit
DVT: Deep vein thrombosis
FEU: Fibrinogen Equivalent Unit
NPV: Negative predictive value
PE: Pulmonary embolism
SLICE: Significance of Pulmonary Embolism in COPD Exacerbations
VTE: Venous thromboembolism
